# Resistance to Antiplatelet Therapy Is Associated With Symptoms of Cerebral Ischemia in Carotid Artery Disease

**DOI:** 10.1177/1538574420947235

**Published:** 2020-08-28

**Authors:** Stephen T. E. Ball, Rachael Taylor, Charles N. McCollum

**Affiliations:** 1 Academic Surgery Unit, Institute of Cardiovascular Sciences, 5292 University of Manchester, Manchester, United Kingdom

**Keywords:** Antiplatelet resistance, carotid artery disease, cerebral ischemia, carotid endarterectomy, cerebral emboli, aggregation

## Abstract

**Background:**

Platelet inhibitory therapy is prescribed to prevent arterial thromboembolism in patients with atherosclerotic disease. Although taken by millions of people, around 30% are resistant to the treatment they are being prescribed.

**Aims:**

To determine whether symptoms of cerebral ischemia, or pre-operative cerebral emboli, in patients admitted for a carotid endarterectomy were associated with resistance to aspirin or clopidogrel.

**Methods:**

Venous blood from 133 patients immediately before carotid endarterectomy (CEA) was analyzed for resistance to aspirin and clopidogrel by multiplate impedance aggregometry. The number of emboli/hour entering the ipsilateral middle cerebral artery was counted by transcranial Doppler (TCD) on the day before surgery in 33 of these patients.

**Results:**

Resistance was found in 21 (26.3%) of 100 patients taking aspirin and 14 (42%) of 33 taking clopidogrel. Mean (sd) residual platelet aggregation was significantly higher at 41.9(32) Au in patients who had suffered recent symptoms of cerebral ischemia compared with 30.8(16) Au in asymptomatic patients (p = 0.012). Residual platelet aggregation also correlated significantly with the number of emboli/hour counted by TCD in the ipsilateral middle cerebral artery (r = 0.45, p = 0.009).

**Conclusion:**

Antiplatelet resistance was associated with the frequency of cerebral emboli and recent symptoms of cerebral ischemia in patients with carotid disease. Definitive clinical studies are needed to explore whether testing for antiplatelet resistance should be undertaken routinely in patients starting platelet inhibitory therapy for cardiovascular disease.

## Introduction

Atherosclerotic arterial disease is the single most frequent cause of death worldwide causing 10 million deaths due to ischemic heart disease and 5.5 million deaths due to stroke each year.^1^ Stroke is the leading cause of disability in the UK and third leading cause of death with >150,000 new strokes/year, costing the UK economy £7 billion/year.^2,3^

Atherosclerotic emboli originating from carotid artery disease (CAD) are thought to cause 30% of all ischemic strokes. Antiplatelet therapy has become established as an essential treatment for all patients with carotid disease reducing annual stroke risk by 9% and preventing 20% of strokes in patients with recent symptoms of cerebral ischemia.^4,5^ However, in the U.S. alone, 185,000 recurrent strokes occur each year, with a third occurring in patients receiving antiplatelet therapy.^6^ Aspirin and Clopidogrel impact on platelet activation, adhesion and aggregation via their own distinct mechanisms but share the common clinical endpoint of reducing thromboembolism from atherosclerotic disease.^7^

It is well documented that inflammatory activity relates to the vulnerability of plaques to rupture; potentially this anti-inflammatory effect was thought to be an important benefit of Aspirin in the Physicians Health Study.^8^ Nevertheless, the main benefit of platelet inhibition is almost certainly the prevention of thrombus formation on vulnerable atherosclerotic plaques and subsequent thromboembolism. Following plaque rupture, collagen and vWF are exposed causing platelet adherence and subsequent thrombus formation which then leads to MI or stroke due to arterial occlusion or distal thromboembolism.^9^

Aspirin and Clopidogrel impact on platelet activation and aggregation via their own distinct mechanisms but share the common endpoint of reducing atherosclerotic progression and platelet associated thrombus formation.^7^ Aspirin inhibits the action of COX-1, inhibiting the synthesis of TXA2 and subsequent platelet activation.^10^ Since platelets are anucleate, enzymatic activity cannot be restored hence they remain inactive for the remainder of their life cycle.^10^

Clopidogrel is an Adenosine Diphosphate receptor antagonist, blocking ADP P2Y12 receptors on the surface, thereby stopping activation of P2Y1 receptors and subsequent platelet aggregation.^11^

Emboli can be detected in the ipsilateral middle cerebral artery in 40% of patients with symptomatic carotid disease. The presence of emboli within 48 hours of stroke is associated with an increased risk of recurrent cerebral ischemic events.^12^ In patients with asymptomatic carotid disease, the presence of cerebral emboli independently predicts 2 year stroke risk.^13,14^ These studies clearly demonstrated the importance of platelet-inhibitory therapy.

The role of platelet-inhibitory therapy in cardiovascular disease has been confirmed by several major clinical trials with the largest recruiting patients with symptomatic arterial disease in any territory (cerebrovascular, coronary and peripheral artery).^15-20^ The Antithrombotic Trialists Collaboration meta-analysis, including 135,000 patients with symptomatic arterial disease, reported a 25% reduction in cardiovascular events in patients taking a range of platelet inhibitory drugs, with Aspirin the most widely studied.^5^

Antiplatelet resistance has been classified as “laboratory” or “clinical.” “Laboratory” antiplatelet resistance is usually measured as continued platelet activity despite platelet inhibitory therapy.^6^ True aspirin resistance occurs at a biochemical and genetic level through polymorphisms of the COX-1/2 genes and thromboxane synthase. For clopidogrel resistance these polymorphisms occur in the cytochrome P450 family.^6^ “Clinical” antiplatelet resistance is defined as treatment failure; cardiovascular events occurring despite antiplatelet therapy, however this may be an oversimplification as strokes can be caused by other pathologies that antiplatelet therapy will not affect such as embolic phenomena secondary to atrial fibrillation. In either event, failures of compliance can lead to “resistance” and true resistance demands confirmation that the relevant drug was taken.

Since aspirin resistance was first reported in the 1980s,^21^ resistance to antiplatelet therapy has been widely reported. In stroke patients taking aspirin for secondary prevention 40% of those resistant to aspirin therapy experienced a serious vascular event within 2 years compared with only 4.4% of aspirin responders.^22^ Testing patients for resistance to platelet inhibitory therapy has not been widely adopted despite evidence that this should be considered.

## Aims

As part of a major study on the importance of carotid plaque volume (CPV) as a cause of cerebral ischemia,^23^ we also explored of the role of resistance to aspirin and clopidogrel in patients admitted for carotid endarterectomy. Our aim was to determine whether resistance to platelet-inhibitory therapy related to the number of cerebral emboli detected in the ipsilateral middle cerebral artery (MCA) by TCD pre-operatively and recent symptoms of cerebral ischemia.

## Methods

*Patients.* All patients undergoing carotid endarterectomy at the University Hospital of South Manchester over an 18 month period were invited to participate. Ethical approval from the National Research Ethics Service North West Committee (11/NW/0308) was obtained with informed consent in writing from all patients. A detailed past medical history was taken and our exclusion criteria were; i) non-compliance with antiplatelet therapy, ii) atrial fibrillation, iii) concomitant use of non-steroidal anti-inflammatories (NSAIDs) within the last 3 months, iv) unable to give informed consent and v) a diagnosis of, or receiving treatment for, cancer within the previous 12 months. Patients were classified as symptomatic or asymptomatic depending on whether they had suffered symptoms of cerebral ischemia within the previous 6 months.

*Measurement of antiplatelet resistance.* Antiplatelet resistance was measured using multiplate impedance aggregometry, a robust and reproducible technique.^24,25^ The electrical impedance generated by platelet aggregation following the administration of a platelet agonist was measured to evaluate residual platelet function.^26^ Multiplate analyzers are also widely used for the measurement of intraoperative platelet function during cardiac surgery.^25,26^

Venous blood (3 ml) was taken preoperatively by atraumatic puncture using an 18 gauge needle into a double wall Hirudin blood tube and gently inverted 3 times to ensure adequate mixing with the anticoagulant. These samples, at room temperature, were transported by hand to avoid excessive shaking and analyzed 90 minutes post venepuncture. 300ul of blood was pipetted into each multiplate test cell and diluted 1:1 with 0.9% saline before being incubated for 3 minutes. 10ul of each agonist was then added to individual cells; Arachidonic Acid, ADP and Thrombin Receptor Activating Peptide (TRAP). Aggregation over 6 minutes was recorded in aggregation units (Au) producing an aggregation curve plotted against time. Patients were classed as resistant to Aspirin or Clopidogrel if the area under the curve was greater than 40Au or 47Au respectively.^27,28^

*Transcranial Doppler.* In a subgroup of 33 patients less than 24 hours before CEA, pre-operative transcranial Doppler (TCD) insonation of the ipsilateral middle cerebral artery using a 2-MHz pulsed-wave Doppler probe (Acuson Multiprobe JH-6007 TCD) was used to count micro-emboli over 1 hour. Micro-embolic signals, defined as transient, unidirectional signals occurring within the Doppler spectrum, at least 3 dB higher than the background blood flow and lasting <300 msec, were counted by 2 trained and blinded observers using the 1995 International Consensus Criteria.^29^

*Carotid Plaque Volume.* The volume of the endarterectomised plaque was measured immediately following carotid surgery using a validated water suspension technique, which has been shown to be accurate and reproducible.^30^ The reliability of this method was also confirmed in our major study reporting the association between CPV and recent symptoms of cerebral ischemia.^23^

*Statistics.* Bivariate associations between antiplatelet resistance and gender, diabetes, hypertension, hyper-cholesterolaemia, rheumatoid arthritis, ischemic heart disease, previous myocardial infarct and smoking status were assessed with the use of Chi-Squared and Fishers Exact test for categorical variables and Independent t-tests for continuous variables. To assess the relationship between continuous variables, Pearson Correlation Coefficient and Spearman’s rho were used for parametric and non-parametric data respectively. Histograms and normality plots were used to assess normality. All statistical analysis was performed using SPSS version 22 with p < 0.05 considered statistically significant.

## Results

Of 133 patients undergoing CEA, 102 (76%) had suffered recent symptoms of cerebral ischemia and 31 were asymptomatic (Table 1). Thirty-three of the 133 (21 symptomatic and 12 asymptomatic) also underwent TCD insonation of the ipsilateral middle cerebral artery to count cerebral emboli over 1 hour.

**Table 1.** Carotid Endarterectomy Patient Characteristics According to Response to Platelet Inhibitory Therapy.

	Resistant (n = 35)	Responder (n = 98)	*p-value*
Gender M: F	23:12	71:27	0.452^*^
Diabetes	6 (17)	8 (8)	0.848^*^
Hypertension	27 (77)	43 (44)	0.018^*^
Hypercholesterolaemia	26 (74)	48 (49)	0.140^*^
Rheumatoid Arthritis	2 (6)	3 (3)	0.648^Ŧ^
IHD	11 (31)	11 (11)	0.248*
Previous MI	6 (17)	7 (7)	0.375^Ŧ^
Current smoker	10 (29)	14 (14)	0.773*
Statin therapy	29 (83)	78 (80)	0.976^Ŧ^
Asymptomatic	5 (14)	26 (26.5)	0.461^Ŧ^
Stroke	9 (26)	26 (26.5)	
TIA	17 (49)	36 (37)	
Amarousis Fugax	4 (11)	10 (10)	

Values in paratheses are percentages unless otherwise stated. *Chi-square, ^Ŧ^ Fishers Exact

Of the 133 CEA patients, 100 were taking Aspirin (75%) and 33 Clopidogrel (25%). No patients were taking dual antiplatelet therapy as it was clinical policy to stop one agent before CEA. The mean age (range) was 69 (47-85) with 94 (91%) men. Resistance to platelet inhibitory therapy was found in a 21 (21%) of the 100 patients taking aspirin and 14 (42%) of the 33 on clopidogrel; an overall frequency of resistance of 35 (26.3%) in the 133 patients.

**Figure 1.**
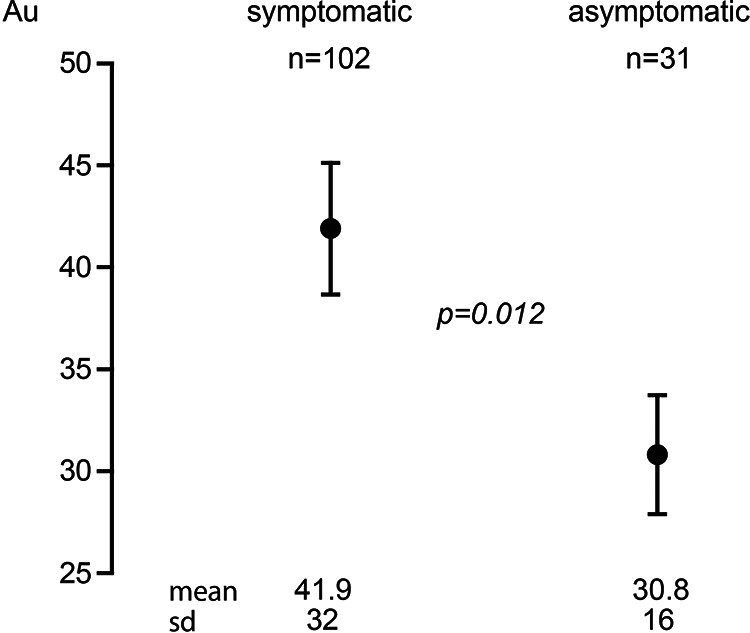
Mean (SD) residual platelet aggregation was significantly higher at 41.9(32)Au in the 102 CEA. Patients with symptoms of cerebral ischemia compared with 30.8(16)Au in the 31 asymptomatic patients (p = 0.012).

Mean (sd) residual platelet activity measured as aggregation units (Au) was significantly higher in the 102 symptomatic patients at 41.9(32) Au compared with 30.8(16)Au in the 31 asymptomatic patients (p = 0.012) (Figure 1). This significance remained following adjustment for diabetes, hypertension and hypercholesterolemia (p = 0.008). There was no significant difference in residual platelet function between patients with TIA, stroke, or amarousis fugax with mean (sd) aggregation of 42.8(32.9) Au for the 53 TIA patients, 42.1(31.6) Au for the 35 stroke patients and 37.7(31.2) Au for the 13 amarousis fugax patients (p = 0.870). When analyzing the type of antiplatelet agent being used, significance remained for those taking clopidogrel; mean platelet aggregation was significantly higher in symptomatic patients at 51.3(29)Au compared with 37.2(8)Au in asymptomatic patients (p = 0.038). However, the significance in the group taking Aspirin was lost (p = 0.065).

**Figure 2.**
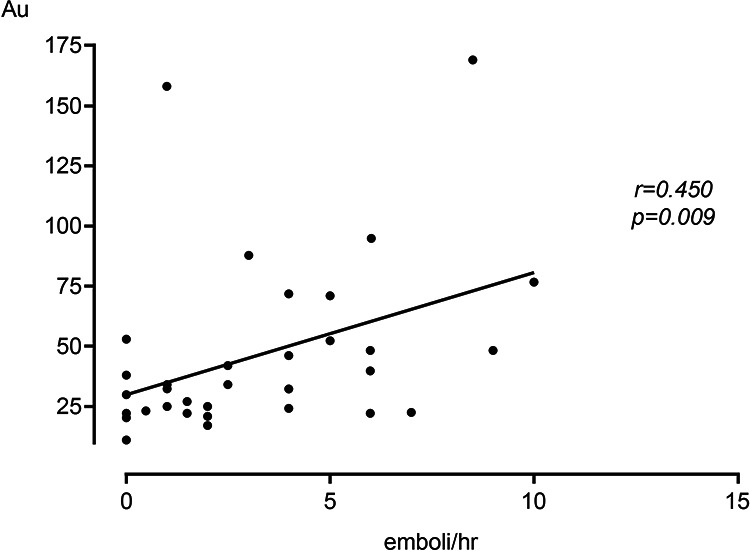
There was a significant correlation of r = 0.45 (p = 0.009) between residual platelet aggregation and the number of cerebral emboli in the ipsilateral middle cerebral artery detected by TCD before CEA in 33, patients.

Of the 33 patients undergoing preoperative TCD to detect cerebral emboli, the 13 patients with antiplatelet resistance had significantly more cerebral emboli at 5.19 ± 2.93/hr compared with just 1.93 ± 1.99/hr in the responders (p = 0.002). This significance remained following adjustment for hypertension (p = 0.004). There was a significant positive correlation between the frequency of cerebral emboli and residual platelet aggregation (r = 0.450, p = 0.009). There was also a significant but weak correlation between the number of cerebral emboli/hour and CPV (r = 0.36, p = 0.045) (Figure 2). When analyzing antiplatelet agents separately, the significance remained. Patients demonstrating resistance to clopidogrel had significantly more emboli at 5.75 ± 2.5/hr compared with 1.25 ± 1.04/hr in responders (p = 0.016). There was also a significant positive correlation between platelet aggregation and frequency of cerebral emboli in those receiving clopidogrel (r = 0.738, p = 0.032). Patients demonstrating resistance to Aspirin also had significantly more emboli at 4.94 ± 3.21/hr compared to 2.09 ± 2.15/hr in responders (p = 0.014). There was also a significant positive correlation between platelet aggregation and frequency of cerebral emboli in those receiving aspirin (r = 0.429, p = 0.037).

Hypertension, but none of the other cardiovascular risk factors, was significantly associated with antiplatelet resistance (p = 0.018) (Table 1). Despite this association between the diagnosis of hypertension and antiplatelet resistance, there was no correlation between the pre-operative systolic blood pressure and residual platelet aggregation (r = −0.131, p = 0.180). There was also no correlation between residual platelet aggregation and patient’s age, BMI or pre-operative blood markers.

Mean (sd) CPV in patients resistant to antiplatelet therapy tended to be greater at 1.03(0.47)cm^3^ compared with 0.95(0.46)cm^3^ in responders, but this small difference did not approach statistical significance (p = 0.371). Nor did CPV correlate with residual platelet aggregation (*r* = 0.072, P = 0.438).

## Discussion

We found a clear link between resistance to platelet inhibitory therapy and both the number of cerebral emboli detected in the middle cerebral artery by TCD before CEA and recent symptoms of cerebral ischemia in patients with carotid artery disease. An association between resistance to antiplatelet therapy and micro-embolic signals in the middle cerebral artery has been reported previously.^31^ In addition, embolic signals in the ipsilateral middle cerebral artery detected in 40% of patients with symptomatic carotid disease were associated with subsequent stroke risk.^12,32^

The association between antiplatelet ressistance and hypertension has also been reported previously suggesting platelet reactivity caused by increased arterial tone, shear stress and endothelial dysfunction in hypertensive patients.^33,34^ Nifedipine, verapamil and diltiazem have all been reported to have a platelet inhibitory effect.^35^ The number of patients on each antihypertensive medication in this study were too small to explore this possibility. We could not confirm a previously reported association between resistance to antiplatelet therapy and systolic blood pressure.^34^

The finding of an overall prevalence of antiplatelet resistance of 26.3% is in line with the published literature, although the published prevalence varies widely between 5.5%-61% for aspirin and 4-30% for clopidogrel.^6,36^ This may be due to the number of different platelet function assays being used; Harrison et al. explored antiplatelet resistance in TIA and ischemic stroke patients using 3 methods: Aspirin resistance was found in 17% of patients using the Verify Now-Aspirin Assay, 22% using the PFA-100 assay, 5% using optical aggregometry.^26,37^ Multiplate impedance aggregometry has been reported to produce some of the most robust and consistently reproducible data.^24-26^

Clinical drug resistance can be due to poor patient compliance, inadequate dosage, drug interactions or increased platelet turnover.^6,38,39^ Non-compliance is thought to be frequent and possibly the most common cause of cardiovascular events; up to 40% of patients with cardiovascular disease do not comply with their aspirin therapy.^6^

The main limitation to this study was the relatively small proportion of our patients undergoing TCD studies for cerebral emboli. This was due to the length of time required for TCD to be performed and a change in unit policy to perform CEAs first on the list. We were also reliant on the patient being accurate regard their compliance to medication. Also, as discussed previously, the wide range of reported antiplatelet resistance is secondary to the number of assays available to test platelet function. While impedance aggregometry is thought to be the most robust method, it is still not wholly reproducible. Hence, given the poor reproducibility, it cannot be fully implemented into clinical practice and maybe genetic testing to detect cellular defects should be sought. Our study would also have been strengthened if we had taken fasting blood levels to explore whether resistance to platelet inhibitory therapy was associated with fasting lipids or HbA1C.

Due to the small numbers involved it is not possible to make any meaningful analysis of the individual antiplatelet agent and compare the outcomes of aspirin resistance to clopidogrel resistance. The effect duration of treatment has on platelet aggregation was not analyzed and needs further attention in future studies given a progressive decline of platelet inhibition has been reported in patients on long term aspirin despite being initially responsive.^40,41^ Both these factors could be addressed in further, larger scale studies.

There has been little research on resistance to antiplatelet therapy in stroke patients. In a study of 281 patients with cerebrovascular disease, aspirin resistance was associated with poor clinical outcomes but was not an independent risk factor for future ischemic events.^42^ However, a meta-analysis including 6450 patients reported that aspirin resistance was significantly associated with CV events despite platelet inhibitory therapy.^43^

This study shows for the first time that resistance to platelet inhibitory therapy was associated with recent symptoms of cerebral ischemia in patients with carotid disease. It also confirms previous studies; i) showing that cerebral emboli were more frequent in patients with antiplatelet resistance and ii) reporting that platelet inhibitory therapy inhibits the number of cerebral emboli in patients with carotid disease.^13^ As microemboli are recognized to be risk factors for both TIA and future stroke, these results tend to confirm that resistance to platelet inhibitory therapy may be a risk factor for stroke in patients with carotid disease. These results also have important implications to the over 7 million people with symptomatic atherosclerotic disease in the UK alone, raising the possibility that around 2 million of these are taking medication that may not be fully effective. These studies emphasize the need to prioritize research on whether all patients with cardiovascular disease should be tested for resistance to the antiplatelet therapy being prescribed.
